# Analyse des données nationales de surveillance de la lèpre en Mauritanie de 2009 à 2019

**DOI:** 10.48327/mtsi.v3i2.2023.361

**Published:** 2023-04-20

**Authors:** Boushab Mohamed BOUSHAB, Pauline Kiswendsida YANOGO, Djibril BARRY, Mohamedou Hmeind MAHAM, Abdallahi Mohamed Kheirou TRAORÉ, Elhadj Malick KANE, Leonardo BASCO, Nicolas MEDA

**Affiliations:** 1 Médecine interne et maladies infectieuses, Centre hospitalier de Kiffa, Assaba, Mauritanie; 2 Programme national de lutte contre la tuberculose et la lèpre (PNTL), Nouakchott, Mauritanie; 3 Faculté de médecine, Université Joseph Ki-Zerbo, Ouagadougou, Burkina Faso; 4 Épidémiologie de terrain et de laboratoire du Burkina Faso, Université Joseph Ki-Zerbo, Ouagadougou, Burkina Faso; 5 Direction générale des Services de santé des Forces armées et de sécurité, Nouakchott, Mauritanie; 6 Aix-Marseille Université, IRD, AP-HM, SSA, VITROME, Marseille, France; 7 IHU-Méditerranée Infection, Marseille, France

**Keywords:** Lèpre, Épidémiologie, Programme de lutte, Mauritanie, Afrique du Nord-Ouest, Leprosy, Epidemiology, Disease control programme, Mauritania, Northwest Africa

## Abstract

**Introduction:**

La lèpre est une maladie infectieuse chronique qui touche principalement la peau, les muqueuses et le système nerveux périphérique. Son élimination en tant que problème de santé publique semble entraîner sa méconnaissance et par conséquent un risque de diagnostic tardif. Une analyse des données de surveillance de la lèpre en Mauritanie a été conduite pour déterminer les tendances épidémiologiques et les formes cliniques des cas notifiés.

**Matériel et méthode:**

L’étude rétrospective a porté sur les relevés épidémiologiques de la lèpre en Mauritanie de 2009 à 2019. Le diagnostic de la lèpre a été posé sur la base des critères diagnostiques de l'Organisation mondiale de la Santé (OMS). Les données ont été analysées avec Epi Info 7.2.5.0.

**Résultats:**

Sur les 11 années, 164 cas ont été notifiés. Parmi ces derniers, 96/164 (58,5%) étaient des hommes et 68/164 (41,5%) des femmes avec un sex-ratio de 1,4. L’âge moyen (± écart-type) était de 44,0 ± 17,1 ans [extrêmes, 9 – 86 ans] et la médiane 45 ans [intervalle interquartile, 32,5; 57,5]. Il y avait 9/164 (5,5%) d'enfants de moins de 16 ans. Les wilayas (ou « gouvernorats ») de Nouakchott étaient les plus endémiques. La forme multibacillaire (MB) représentait 109/164 (66,5%) des cas. L'incidence annuelle moyenne était de 0,3 cas/100 000 habitants pour la forme MB et 0,1 cas/100 000 pour la forme paucibacillaire (PB). Tous les cas notifiés ont été traités par polychimiothérapie.

**Conclusion:**

Les résultats de la surveillance de la lèpre montrent une persistance de cette maladie en Mauritanie. Il est nécessaire de relancer à tous les niveaux les services de lèpre afin de continuer à réduire la morbidité liée à cette maladie, voire l’éliminer.

## Abstract (en)

**Introduction:**

Leprosy is a chronic infectious disease that mainly affects the skin, mucous membranes and the peripheral nervous system. Its elimination as a public health problem seems to lead to its ignorance and therefore to a risk of late diagnosis. An analysis of leprosy surveillance data in Mauritania was conducted to determine epidemiological trends and clinical forms of reported cases.

**Material and method:**

The retrospective study was based on the epidemiological records of leprosy in Mauritania from 2009 to 2019. The diagnosis of leprosy was made on the basis of the diagnostic criteria of the World Health Organization (WHO). Data were analyzed using Epi Info version 7.2.5.0. The frequencies, proportions, and rates were calculated.

**Results:**

Over the past 11 years, 164 cases have been notified. Among the notified cases, 96/164 (58.5%) were males and 68/164 (41.5%) females, with a sex ratio of 1.4. The mean age (± standard deviation) was 44.0 ± 17.1 years [range, 9 – 86 years], and the median was 45 years [interquartile range, 32.5; 57.5]. Children under the age of 16 accounted for 9/164 (5.5%). The wilayas (i.e. “regions”) of Nouakchott were the most endemic regions in the country. The multibacillary form (MB) represented 109/164 (66.5%) cases among the observed clinical forms. The average annual incidence was 0.3 case/100,000 population for MB and 0.1 case/100,000 for PB (paucibacillary). All reported cases were treated with multidrug therapy.

**Conclusion:**

The results of leprosy surveillance showed a persistence of this disease in Mauritania. It is necessary to relaunch leprosy services at all levels in order to continue to reduce the morbidity associated with this disease, and eventually eliminate it from the country.

## Introduction

La lèpre ou maladie de Hansen est une infection chronique causée par *Mycobacterium leprae* ou *Mycobacterium lepromatosis.* C'est une maladie tropicale négligée (MTN) encore présente dans plus de 120 pays sous forme endémique, avec plus de 200 000 nouveaux cas signalés chaque année [5, 7]. C'est aussi la maladie infectieuse qui est à l'origine du plus grand nombre de difformités physiques. La stigmatisation des personnes atteintes de lèpre demeure un obstacle à la détection précoce de la maladie et des cas de discrimination à l’égard des malades continuent d’être constatés. La lèpre est sous-diagnostiquée parce qu'elle n'est plus enseignée dans les programmes médicaux. L'attention s'est déplacée de la lèpre vers la tuberculose et les infections par le virus de l'immunodéficience humaine (VIH) à la fin du xx^e^ siècle, au cours duquel le programme de lutte contre la lèpre de l'Organisation mondiale de la Santé (OMS) a été réduit avec la conviction que la lèpre était pratiquement éliminée [1]. L'objectif d'un taux d'incidence inférieur à 1 cas/10 000 habitants/an était atteint en 2000. Cependant, en 2005, la lèpre restait endémique dans 6 pays. La Mauritanie, comme d'autres pays où la lèpre sévit, a mis en place un Programme national de lutte contre la tuberculose et la lèpre (PNTL) en 1988. Malgré les mesures prises, des cas de lèpre y sont encore notifiés. Cette étude a pour objectif d'analyser les données de surveillance nationale de la lèpre en Mauritanie afin de calculer son taux d'incidence et de formuler des recommandations pour l'amélioration des performances de ce programme.

## Matériel et Méthode

La République islamique de Mauritanie comprend un territoire de plus de 1 million de km^2^ avec une faible densité de population (environ 5 millions d'habitants en 2020) inégalement répartie. Sa position entre le 15^e^ et le 27^e^ degré de latitude nord en fait un pays de contact et de transition entre le désert saharien (70 % du territoire) au nord et les steppes sahéliennes au sud.

Cette étude rétrospective porte sur les cas de lèpre enregistrés dans la base de données du PNTL du ministère de la santé de 2009 à 2019. Le diagnostic de la lèpre a été posé sur la base des critères diagnostiques de l'OMS:

La lèpre est dite paucibacillaire (PB) lorsqu'il existe 2 à 5 lésions cutanées anesthésiques à distribution asymétrique et une atteinte neurologique d'un seul nerf.

La lèpre est dite multibacillaire (MB) lorsqu'il existe plus de 5 lésions cutanées hypoesthésiques, de distribution plus symétrique avec atteinte de plusieurs nerfs [7].

Le diagnostic de la lèpre en Mauritanie est passif: le patient se présente dans une structure sanitaire en cas de signes évocateurs. Les patients diagnostiqués sont ensuite adressés au Centre de diagnostic et de traitement (CDT) pour une prise en charge. Les données épidémiologiques de base ont été notées pour chaque cas: âge, sexe, wilaya de provenance et forme de la maladie. Les données ont été analysées à l'aide du logiciel Epi Info 7.2.5.0. Notre étude a été réalisée avec l'autorisation des autorités sanitaires de la Mauritanie pour l'acquisition et l'analyse de la base de données. Les noms et prénoms des cas ont été anonymisés pour garantir la confidentialité.

## Résultats

**Figure 1:**
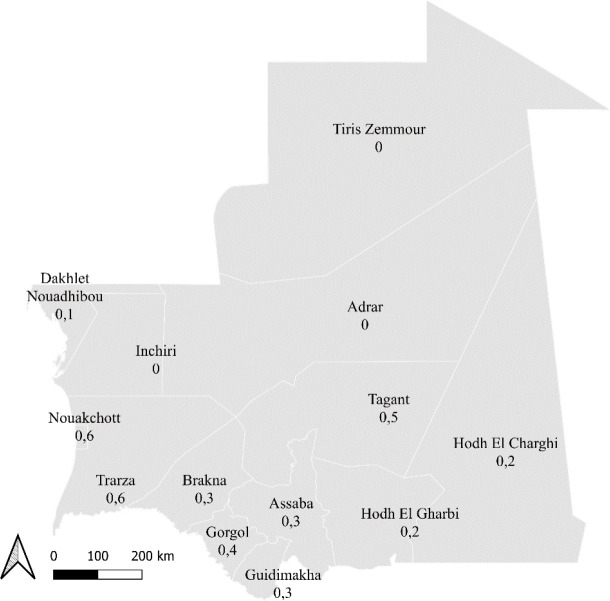
Incidence annuelle pour 100 000 habitants des cas de lèpre selon les wilayas (gouvernorats). Les données des trois wilayas de Nouakchott ont été regroupées. Données sur la population: 2018, www.populationdata.net/pays/mauritanie/ Annual incidence of leprosy cases for 100.000 inhabitants according to the wilayas. The data of the three wilayas of Nouakchott are totaled. Data on population: 2018, www.populationdata.net/pays/mauritanie/

Sur une période de 11 ans, allant de janvier 2009 à décembre 2019, un total de 164 cas de lèpre ont été notifiés par le PNTL. Parmi les cas notifiés, 96 (58,5%) étaient des hommes et 68 (41,5%) des femmes avec un sex-ratio de 1,4. L’âge moyen (± écart-type) était de 44,0 ± 17,1 ans (extrêmes, 9 – 86 ans) et l’âge médian de 45 ans [intervalle interquartile, 32,5; 57,5] (Tableau I). Les tranches d’âge les plus concernées étaient les 46-60 ans (30,5 % des cas), suivis des 31-45 ans (28,7 % des cas). Les moins de 16 ans étaient 9 (5,5%). Parmi les 15 wilayas (gouvernorats) que compte le pays, le plus grand nombre de cas a été détecté dans les wilayas de Nouakchott (77/164, soit 47 % cas) (Fig. 1). Depuis 2020, la ville de Nouakchott est divisée en trois wilayas: Nouakchott-Nord (26 cas), Nouakchott-Ouest (6 cas) et Nouakchott-Sud (21 cas), avec 24 cas sans région précisée. Aucun cas n'a été rapporté de 3 wilayas: Tiris Zemmour, Adrar et Inchiri. Ces wilayas, situées en plein Sahara, sont peu peuplées et mal desservies par les services de santé.

**Tableau I:** Répartition des cas de lèpre selon les tranches d’âge, le sexe et la wilaya de provenance Distribution of leprosy cases according to age group, sex and geographic origin

Caractéristiques socio-démographiques	Effectif	Fréquence (%)
Tranche d’âge (ans)		
0-15	9	5,5
16-30	29	17,7
31-45	47	28,7
46-60	50	30,5
61-75	26	15,8
76-90	3	1,8
Sexe		
masculin	96	58,5
féminin	68	41,5
Wilaya de provenance		
Nouakchott-Nord	26	16
Nouakchott-Ouest	6	4
Noukchott-Sud	21	13
Nouakchott*	24	15
Trarza	19	12
Gorgol	17	10
Hodh El Charghi	12	7
Assaba	11	7
Brakna	9	5
Hodh El Gharbi	6	4
Guidimakha	6	4
Tagant	5	3
Dakhlet Nouadhibou	2	1
Tiris Zemmour	0	0
Adrar	0	0
Inchiri	0	0

* La ville de Nouakchott est composée de trois wilayas, à savoir Nouakchott-Nord, Nouakchott-Ouest et Nouakchott-Sud. Sur 77 cas de lèpre notifiés à Nouakchott, la provenance exacte de 24 (31%) n'a pas été précisée.

**Figure 2:**
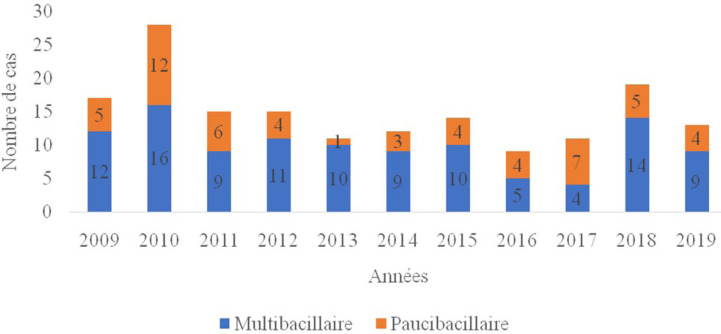
Répartition des cas de lèpre (multibacillaire et paucibacillaire) selon les années Distribution of leprosy cases (multibacillary and paucibacillary) depending on the year

Sur le plan clinique, 109 cas (66,5%) avaient une forme MB et 55 cas (33,5%) une forme PB (Fig. 2). Parmi les patients MB, 49/109 (45%) résidaient à Nouakchott.

**Figure 3:**
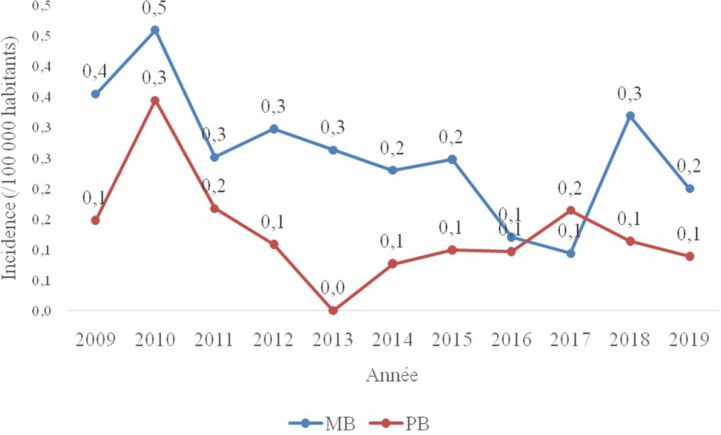
Incidence annuelle des formes cliniques de la lèpre en Mauritanie de 2009 à 2019 (nombre de cas/100 000 habitants/an). MB, multibacillaire; PB, paucibacillaire Annual incidence of clinical forms of leprosy in Mauritania from 2009 to 2019 (number of cases/10,000 inhabitants/year). MB, multibacillary; PB, paucibacillary

L'incidence annuelle nationale de l'ensemble des cas entre 2009 et 2019 était variable, soit en moyenne de 0,3 cas pour 100 000 habitants pour la forme MB et 0,1 cas/100 000 pour la PB (Fig. 3). Il n'y avait pas de différence statistiquement significative (*p* > 0,05) entre le sexe, la tranche d’âge, la provenance et la forme clinique.

Tous les patients ont été traités par polychimiothérapie (PCT): dapsone, rifampicine et clofazimine pour une durée de 12 mois pour les formes MB; dapsone et rifampicine pour une durée de 6 mois dans les formes PB. La prise de la PCT se faisait sous la supervision de l'agent de santé de la moughataa (c'est-à-dire le département).

## Discussion

Il s'agit de la première étude utilisant la base de données nationales du PNLT de 2009 à 2019. Les données sont considérées comme exhaustives pour les patients ayant bénéficié d'une prise en charge. Cette étude met en évidence une prédominance masculine. Ceci confirme les données de la littérature [2, 3, 9]. L’âge inférieur à 16 ans était observé dans 5,5 % des cas. Cette proportion des enfants est classiquement considérée comme le reflet d'une importante transmission de la maladie. En pays d'endémie, la lèpre se manifeste durant la première moitié de vie, la contamination se faisant dans le jeune âge alors qu'elle surviendrait plus tardivement dans les zones où la maladie est marginale [2]. Nos données relatives aux cas de lèpre chez les enfants suggèrent que la transmission de la lèpre reste active, voire mal maîtrisée.

Quarante-sept pour cent de cas provenaient des wilayas de Nouakchott (1,5 million d'habitants soit 30 % de la population). Cette prédominance est liée d'une part à la présence de l'unique service de dermatologie, service de référence du Centre hospitalier national de Nouakchott, d'autre part au flux migratoire vers la grande ville. La classification clinique de l'OMS utilisée dans les structures de santé pour le diagnostic des formes ne permet pas de définir les formes indéterminées. Par ailleurs, aucun cas de réaction réverse (c'est-à-dire, un effet indésirable qui peut survenir pendant le traitement de la lèpre) n'a été rapporté au cours de la période d’étude. Cela est dû, d'une part, au fait que les cas de lèpre sont enregistrés au début du traitement et, d'autre part, au fait que les réactions réverses sont souvent diagnostiquées par des dermatologues, internistes et/ou des généralistes qualifiés et ne sont pas rapportées aux personnels référents de la lèpre dans les moughataas sanitaires pour y être notifiées. Ceci illustre les difficultés de diagnostic et de notification des cas à l'intérieur du pays. Des constatations semblables ont été faites au Togo [9].

L’étude montre une nette prédominance des formes MB. Il en est de même dans plusieurs pays où la lèpre a été déclarée ne plus constituer un problème de santé publique [2, 4, 6, 8]. La forte proportion des formes MB augmente les risques de contagiosité de la maladie. La durée du traitement plus longue nécessaire pour les formes MB peut conduire à l'abandon du traitement par les patients et à une augmentation des perdus de vue, et donc contribuer à la propagation de la maladie puis à la survenue de formes résistantes [7]. Parmi les cas atteints de forme MB, 45 % résidaient à Nouakchott où la densité démographique est plus élevée que pour d'autres villes mauritaniennes. Bien que ces cas notifiés à Nouakchott puissent être surestimés à cause de l'exode rural et du sous-dépistage hors de la capitale, le risque de transmission de la lèpre dans la capitale serait plus élevé que dans un village peu peuplé en raison de la promiscuité.

Compte tenu du caractère rétrospectif, il est difficile d'expliquer les variations de taux d'incidence observés entre 2009 et 2019 en Mauritanie. Cependant, elles pourraient être dues à la faiblesse du système de prise en charge des cas de lèpre pendant cette période. Le dépistage passif de la lèpre, les lacunes de notification, les difficultés d'accès des patients aux structures de soins, les pratiques de l'automédication et l'absence de sensibilisation communautaire pourraient expliquer ces variations de l'incidence d'une année à une autre. L'affaiblissement du système immunitaire, dû à l'absence d'eau potable et/ou à l'absence de mesures d'hygiène appropriées et dû à une alimentation de mauvaise qualité, est reconnu depuis longtemps comme un des déterminants importants pour la survenue de la lèpre [7]. Sur le plan thérapeutique, les recommandations de l'OMS ont été appliquées à tous les patients. Aucun cas d'infirmité, de résistance au traitement, de rechute ou de perdu de vue n'a été mentionné par le PNTL, ce qui pourrait témoigner d'un manque de complétude des données.

Bien que la lèpre ne soit plus un problème majeur de santé publique en Mauritanie, il est important d'amplifier les activités de surveillance et d’élimination. La prédominance des formes MB traduit les retards de diagnostic et la probable sous-évaluation de l'incidence réelle.

Une des limites de cette étude est son caractère rétrospectif qui ne signale que les cas notifiés par le PNLT. Les données sur les cas contacts, le prélèvement des frottis, le retraitement (c'est-à-dire une reprise de traitement médicamenteux en cas de réapparition des symptômes de la maladie ou de réaction de réversion en cas d'interruption du traitement précédent, d'inefficacité du traitement initial ou de résistance des bacilles de la lèpre), la résistance au traitement et les réactions lépreuses ne sont pas systématiquement signalés. Cela est dû, d'une part, au fait que les cas sont enregistrés au début du traitement et, d'autre part, que les personnels référents du PNLT dans les CDT ne rapportent pas ces données souvent diagnostiquées par des spécialistes (dermatologues, médecins internistes, infectiologues ou généralistes qualifiés). Ceci est d'autant plus important qu'elles renseignent sur la qualité du dépistage, du traitement et du suivi des cas.

Pour remédier à cela, il faudrait envisager une étude des cas de lèpre diagnostiqués, permettant alors d'obtenir tous les paramètres cliniques et biologiques importants en vue d'une analyse comparative détaillée et précise. À cet effet, nous recommandons de redynamiser les services de lèpre à tous les niveaux. Les stratégies de communication doivent être développées en direction de la population pour obtenir adhésion et collaboration.

## Conclusion

La lèpre sévit encore en Mauritanie. Sa transmission persiste au sein des communautés. La prédominance de la forme MB traduit les retards de diagnostic. Aussi importe-t-il de renforcer les efforts de prévention et de traitement de la lèpre en Mauritanie, en mettant en place des campagnes d'information, d’éducation et de communication pour une consultation précoce dès l'apparition d'une lésion cutanée hypochromique, en développant des équipes mobiles pour améliorer la détection des nouveaux cas, en renforçant les capacités des agents communautaires de santé pour le diagnostic, en capitalisant les acquis du passé pour améliorer le système de surveillance, en révisant les outils de collecte des données pour prendre en compte les nouveaux besoins d'information, et en promouvant les activités de prévention des invalidités et de réadaptation physique.

## Contribution Des Auteurs

Boushab Mohamed BOUSHAB: revue de littérature, rédaction du manuscrit.

Pauline Kiswendsida YANOGO, Djibril BARRY, Mohamedou Hmeind MAHAM: analyse et l'interprétation des données.

Abdallahi Mohamed Kheirou TRAORÉ, Elhadj Malick KANE: collecte des données et correction du style du manuscrit.

Leonardo BASCO: coordination scientifique générale de l’étude, analyse et l'interprétation des données et préparation du manuscrit final.

Nicolas MEDA: responsable de la coordination scientifique globale de l’étude, rédaction du manuscrit et finalisation. Tous les auteurs ont lu et approuvé la version finale du manuscrit.

## Liens D'intérêt

Les auteurs ne déclarent aucun lien d'intérêt.
